# Sulfated vizantin suppresses mucin layer penetration dependent on the flagella motility of *Pseudomonas aeruginosa* PAO1

**DOI:** 10.1371/journal.pone.0206696

**Published:** 2018-11-01

**Authors:** Naoki Hayashi, Yui Furue, Daichi Kai, Noriteru Yamada, Hirofumi Yamamoto, Takashi Nakano, Masataka Oda

**Affiliations:** 1 Department of Microbiology and Infection Control Science, Kyoto Pharmaceutical University, Kyoto, Japan; 2 Department of Chemistry and Functional Molecule, Faculty of Pharmaceutical Sciences, Tokushima Bunri University, Tokushima, Japan; 3 Department of Microbiology and Infection Control, Osaka Medical College, Osaka, Japan; Seconda Universita degli Studi di Napoli, ITALY

## Abstract

*Pseudomonas aeruginosa* is an opportunistic pathogen that causes severe infections, such as pneumonia and bacteremia. Several studies demonstrated that flagellar motility is an important virulence factor for *P*. *aeruginosa* infection. In this study, we determined whether sulfated vizantin affects *P*. *aeruginosa* flagellar motility in the absence of direct antimicrobial activity. We found that 100 μM sulfated vizantin suppressed *P*. *aeruginosa* PAO1 from penetrating through an artificial mucin layer by affecting flagellar motility, although it did not influence growth nor bacterial protease activity. To further clarify the mechanism in which sulfated vizantin suppresses the flagellar motility of *P*. *aeruginosa* PAO1, we examined the effects of sulfated vizantin on the composition of the flagellar filament and mRNA expression of several flagella-related genes, finding that sulfated vizantin did not influence the composition of the flagellar complex (*fliC*, *motA*, and *motB*) in *P*. *aeruginosa* PAO1, but significantly decreased mRNA expression of the chemotaxis-related genes *cheR*1, *cheW*, and *cheZ*. These results indicated that sulfated vizantin is an effective inhibitor of flagellar motility in *P*. *aeruginosa*.

## Introduction

*Pseudomonas aeruginosa* is an opportunistic pathogen that causes severe pneumonia and bacteremia, which are associated with high mortality [1, 2]. Colonization with *P*. *aeruginosa* occurs in critical body organs, such as the lung [3], urinary tract [4], and intestine [5], with potentially fatal results. Mucins, which are high-molecular-weight glycoproteins, can be divided into membrane-bound and secretory forms depending on their location and structure [6, 7]. Numerous microorganisms can penetrate mucous layers using motility and enzymes that degrade mucins in order to reach host epithelial-cell surfaces [6, 7]. *P*. *aeruginosa* can also subvert mucin layers and reach the surface of host epithelial cells [8].

*P*. *aeruginosa* utilizes many kinds of virulence factors, including flagella, type IV pili, types II, III, and VI effectors, biofilm, proteases, and lipopolysaccharides [9–11]. *P*. *aeruginosa* flagellar motility, such as swimming, is derived from rotation of the flagellar filament, which consists of a flagellar filament protein (FliC) polymer powered by a motor complex comprised of MotAB and MotCD [12]. Flagellar filament rotation of *P*. *aeruginosa* toward high levels of nutrients or to escape toxic compounds requires a chemotaxis system [13]. Current studies suggest that *P*. *aeruginosa* has five gene clusters (clusters I–V) involved in chemotaxis, including genes encoding 26 methyl-accepting chemotaxis proteins (MCPs), which are homologous to *Escherichia coli* MCP genes, and 20 chemotaxis genes [13–16]. Among the five gene clusters, clusters I and V (*che*) and cluster II (*che2*) regulate flagella, cluster III is involved in biological functions unrelated to chemotaxis, and cluster IV assembles into a pathway associated with the Pil–Chp operon that controls type IV pilus production and twitching motility in *P*. *aeruginosa* PAO1 [16]. Several animal studies involving infections of the lung, urinary tract, and intestine demonstrated the importance of flagella as a virulence factor in some *P*. *aeruginosa* strains [17–20]. In a mouse pneumonia model of infection, antibody to flagella suppressed the virulence of *P*. *aeruginosa* PAO1 [17]. Additionally, a genetic flagellar mutant (*fliC*) of PAO1 also suppressed virulence in a burned-mouse model of infection [21]. Furthermore, we previously demonstrated that *P*. *aeruginosa* PAO1 can penetrate the mucin layer through flagellar motility and a mucin degrading protease [8, 22]. These findings indicate that compounds attenuating *P*. *aeruginosa* motility could have potential applications toward limiting *P*. *aeruginosa* infection.

*P*. *aeruginosa* is naturally less susceptible to many antibiotics and often develops resistance to various antibiotics during ongoing therapy. In particular, the incidence of multidrug-resistant (MDR) *P*. *aeruginosa*, which is resistant to carbapenems, aminoglycosides, and fluoroquinolones, has increased significantly [23]. This situation is a matter of concern due to its potential for becoming a global health problem, with the Centers for Disease Control and Prevention considering MDR *P*. *aeruginosa* a serious threat to public health (https://www.cdc.gov/drugresistance/biggest_threats.html). In many cases, successful treatment of a patient infected with MDR *P*. *aeruginosa* involves the use of colistin [24]; however, transmissible colistin resistance has emerged and spread worldwide, although its prevalence remains quite low [23–25]. A strategy to overcome this potential global health challenge involves development of new drugs that block bacterial virulence factors.

Vizantin is a derivative of trehalose-6,6'-dimycolate extracted from *Mycobacterium tuberculosis* [26] and that activates the innate immune response through specific binding to the toll-like receptor 4 (TLR4)/myeloid differentiation factor 2 (MD2) protein complex without inducing tumor necrosis factor α production [27]. Because vizantin competes with lipopolysaccharide for TLR4/MD2 binding, it potentially represents a safe therapeutic agent for endotoxemic phenomena, including sepsis-like symptoms and septic shock [28]. Recently, we developed 2,2',3,3',4,4'-hexasulfated-vizantin (sulfated vizantin), which successfully solubilized vizantin (S1 Appendix). This sulfated vizantin induces DNA-based extracellular traps in phagocytic cells and induces phagocytosis of *P*. *aeruginosa* [29]; however, the mechanism associated with this activity, which directly acts on bacteria, remains unclear. In this study, we examined whether sulfated vizantin significantly inhibits *P*. *aeruginosa* flagellar motility in the absence of direct antimicrobial activity. Although sulfated vizantin did not affect the composition of the flagellar filament, we found that it significantly decreased mRNA expression of chemotaxis-related genes that control the flagellar motor complex in *P*. *aeruginosa* PAO1. Therefore, our findings suggest that sulfated vizantin represents an effective inhibitor of flagellar motility in *P*. *aeruginosa*.

## Materials and methods

### Bacterial strains, growth conditions, and sulfated vizantin

*P*. *aeruginosa* wild-type PAO1 [14] and Δ*fliC* [8] were from our laboratory stock strains that were grown on Luria-Bertani (LB) agar plates (Nippon Becton Dickinson Company, Tokyo, Japan) or in LB broth (Nippon Becton Dickinson Company) at 37°C. Vizantin and sulfated vizantin were prepared according to previously reported procedure for their synthesis [29].

### Mucin penetration assay

We performed a mucin penetration assay, as described previously [8,22]. Mucin chambers were prepared by adding 50 μL of 5% bovine submaxillary mucin (Sigma-Aldrich, St. Louis, MO, USA) in Dulbecco’s modified Eagle medium (DMEM) with high glucose (Sigma-Aldrich) to 0.143-cm^2^ membranes of Transwell inserts (3.0-μm pore filter; Corning, Corning, NY, USA). Next, 25 μL of *P*. *aeruginosa* culture [5.0 × 10^4^ colony forming units (CFU)] was dropped onto the top of the artificial mucin layers, and 235 μL DMEM with or without sulfated vizantin (100 μM) was added to the Transwell upper chamber. After a 3 h incubation at 37°C in 5% CO_2_, *P*. *aeruginosa* in the bottom chamber was collected. Appropriate dilutions of *P*. *aeruginosa* were then spread on LB agar plates, incubated at 37°C for 16 h, and CFUs were counted to quantify the bacteria.

### Measurement of bacterial growth

*P*. *aeruginosa* PAO1 was incubated with or without sulfated vizantin (100 μM) in LB broth at 37°C in 5% CO_2_. After incubation at the indicated times, the optical density at 600 nm (OD_600_) was measured using a Varioskan Flash microplate reader (Thermo Fisher Scientific, Waltham, MA, USA). Additionally, to measure the number of viable bacteria, *P*. *aeruginosa* PAO1 was incubated with or without sulfated vizantin (100 μM) in LB broth at 37°C in 5% CO_2_ for 3 h. After incubation, appropriate dilutions of *P*. *aeruginosa* were then spread on LB agar plates, incubated at 37°C for 16 h, and CFUs were counted to quantify the bacteria.

### Swimming assay

We performed a swimming assay as described previously [8]. Sulfated vizantin (100 μM) was added to soft agar containing 0.3% (w/v) bacto agar (Nippon Becton Dickinson Company) at 65°C. *P*. *aeruginosa* with or without sulfated vizantin (100 μM) was spotted on the center of the swimming agar plate, and after incubation at 37°C in 5% CO_2_ for 12 h, the diameter distance (mm) of the swimming zones were measured.

### Protease activity assay

We performed a protease activity assay as described previously [8]. *P*. *aeruginosa* PAO1 was incubated with or without sulfated vizantin (100 μM) in LB broth at 37°C in 5% CO_2_ for 3 h, followed by addition of 0.3% azocasein (Sigma-Aldrich) in a solution containing 50 mM Tris-HCI and 0.5 mM CaCl_2_ (pH 7.5) to the *P*. *aeruginosa* culture. LB broth was used as control, and a negative control assay was performed by using 0.3% azocasein solution with protease inhibitors (Complete; Roche Diagnostics, Basel, Switzerland) according to manufacturer instructions. After incubation at 37°C for 15 min, undigested substrate was precipitated with 10% trichloroacetic acid and removed by centrifugation (10,000*g* for 10 min). The supernatant was mixed with an equal volume of 1 M NaOH, and color development was quantified spectrophotometrically at 440 nm.

### Tracking images of *P*. *aeruginosa* motility

*P*. *aeruginosa* was incubated with or without sulfated vizantin (100 μM) at 37°C in 5% CO_2_ for 3 h. After incubation, bacterial culture was mixed with 0.5% Seakem GTG agarose gel (Cambrex Bio Science, Rockland, ME, USA), loaded onto a slide, and cellular motilities were recorded as a movie using an EVOS microscope with a 100× objective (Thermo Fisher Scientific). Moving distances of *P*. *aeruginosa* PAO1 were tracked using the MTrackJ plugin (https://www.imagescience.org/meijering/software/mtrackj/) developed for ImageJ (https://imagej.nih.gov/ij/; National Institutes of Health, Bethesda, MD, USA). Tracked data were then analyzed to yield bacterial speed.

### Flagellar-filament protein expression

After incubation of *P*. *aeruginosa* in LB broth with or without 100 μM sulfated vizantin at 37°C for 3 h, cells were collected by centrifugation. FliC protein in collected cells was detected by western blot analysis using an anti-FliC rabbit polyclonal antibody (Scrum, Tokyo, Japan) and goat anti-rabbit IgG H&L (Abcam, Cambridge, MA, USA). Bound antibodies were then visualized using the enhanced chemiluminescence prime western blot detection system (GE Healthcare, Waukesha, WI, USA) and scanned with a C-DiGit imaging system (M&S TechnoSystems, Osaka, Japan). Band intensities were measured using ImageJ software (National Institutes of Health) with the magic wand tool where appropriate.

### Surface-flagella enzyme-linked immunosorbent assay (ELISA)

We performed ELISA as described previously [30]. Briefly, *P*. *aeruginosa* was incubated in LB broth with or without 100 μM sulfated vizantin at 37°C for 3 h until bacterial cells in phosphate-buffered saline reached an OD_600_ of 1.0. Serial dilutions of the suspensions were added to 96-well ELISA plates (Thermo Fisher Scientific) for incubation for 2 h at 37°C, and the antigen was fixed with methanol for 30 min. ELISA plates were then washed with Tris-buffered saline-Tween 20 (TBST) prior to the addition of the anti-FliC polyclonal antibody (Scrum). ELISA plates were washed three times with TBST, followed by addition of goat anti-rabbit IgG H&L (Abcam). Detection was performed with TMB-ELISA solution (Thermo Fisher Scientific), and absorbance (450 nm) in the wells was determined using a Varioskan Flash microplate reader (Thermo Fisher Scientific).

### Flagellar-filament observation with Leifson stain

*P*. *aeruginosa* flagella were stained with Leifson stain [31]. After incubating *P*. *aeruginosa* in LB broth with or without 100 μM sulfated vizantin at 37°C for 3 h, the bacterial culture was suspended in 5% formalin and then spotted onto a glass slide. Following fixation for 10 min at room temperature, bacteria were stained with Leifson dye solution (Mutokagaku, Tokyo, Japan) and washed with distilled water. Stained bacteria were analyzed under an IX71 microscope (Olympus, Tokyo, Japan).

### Flagellar-filament observation by transmission electron microscopy (TEM)

After incubating *P*. *aeruginosa* in LB broth with or without 100 μM sulfated vizantin at 37°C for 3 h, the bacterial culture was fixed with an equal amount of 4% glutaraldehyde in 0.1 M phosphate buffer (pH 6.8), the sample was dehydrated in a graded ethanol series and 3-methylbutyl acetate, and dried in a HCP-1 critical-point drying chamber (Hitachi High-Technologies Corp., Tokyo, Japan). The sample was then coated with a platinum layer ~1-nm thick in an E-1030 ion sputter coater (Hitachi High-Technologies Corp.). Samples were examined under a S-5000 scanning electron microscope (Hitachi High-Technologies Corp.).

### Flagella-related gene expression

Semi-quantitative reverse transcription polymerase chain reaction (RT-PCR) was performed as described previously, with minor modifications [30]. *P*. *aeruginosa* PAO1 was incubated with or without sulfated vizantin (100 μM) at 37°C in 5% CO_2_ for 3 h, followed by cell collection by centrifugation at 15,000*g* for 5 min and then lysis with TRI Reagent (Sigma-Aldrich) according to manufacturer instructions. The purified RNA was treated with recombinant DNase I (Takara, Shiga, Japan), and RT-PCR was performed using a PrimeScript One Step RT-PCR kit (v.2; Takara) and primers (Table 1) according to manufacturer instructions. RT-PCR was performed using the following procedure: one cycle at 50°C for 30 min and 94°C for 2 min; 28 cycles of 95°C for 30 s, 55°C for 30 s, and 72°C for 1 min, followed by a final extension at 72°C for 1 min. RT-PCR products were electrophoresed on a 2% agarose gel and observed under ultraviolet illumination after staining with ethidium bromide. The *gyrB* gene was used as an internal control, and band intensities were measured using ImageJ software (National Institutes of Health). Measurement of RT-PCR products was conducted using ImageJ software (National Institutes of Health) by manually selecting each band and using the magic wand tool where appropriate.

**Table 1:** Primers used in this study.

Target gene	Primer	Sequence (5′ → 3′)
*gyrB* (PA0004)	gyrB-RT-F	TGCTGAAGGGGCTGGATGCCGTACGCAAGC
	gyrB-RT-R	TATCCACCGGAATACCGCGTCCATTGTCGC
*fliC* (PA1092)	fliC-RT-F	CTGACCTCGGTGCTGTTCAG
	fliC-RT-R	GAGCGTTGGTAGCGTTTTCC
*motA* (PA4954)	motA-RT-F	CATGCTCTACGAGATCCTCAAC
	motA-RT-R	GGTCATCCGCTCATCCTTC
*motC* (PA1460)	motC-RT-F	GACCTGTTCACCCAGGAAAGTC
	motC-RT-R	CCGATGATGCCGATGGTC
*cheA* (PA1458)	cheA-RT-F	ACTTTGGCGATCATGCCTAC
	cheA-RT-R	CCAGCGCTTGAGATAGAACAG
*cheB* (PA1459)	cheB-RT-F	GATCACCATGGATTACGAGATG
	cheB-RT-R	CGAAATTCTTCGGCAGGTAG
*cheR*1 (PA3348)	cheR1-RT-F	GTCTGCAACGCTATTTCGAC
	cheR1-RT-R	TCGGCGGAGAAATAGATCAG
*cheW* (PA1464)	cheW-RT-F	TCAATGTGATGCAGGTCCAG
	cheW-RT-R	TTGTCCGCCTCGATAATGAC
*cheY* (PA1456)	cheY-RT-F	AACCTCTTGCGGGACTTGG
	cheY-RT-R	TCCAGTCGGTGACGAGAAAG
*cheZ* (PA1457)	cheZ-RT-F	TGATCAAGCGTGTCACCAAG
	cheZ-RT-R	CCACATCCTCTCGTTTTTCC

### Statistical analysis

Statistical analysis, except measurement of bacterial growth, was performed using one-way analysis of variance according to Tukey's test using EZR [32]. Bacterial growth was evaluated using Student’s *t* test at each time point, with relative cell viability analyzed using the same method and EZR [32]. EZR is a modified version of R commander and designed to add statistical functions frequently used in biostatistics. A *P* < 0.05 was considered statistically significant.

## Results

### Sulfated vizantin decreases penetration of *P*. *aeruginosa* through the mucin layer

**Fig 1:**
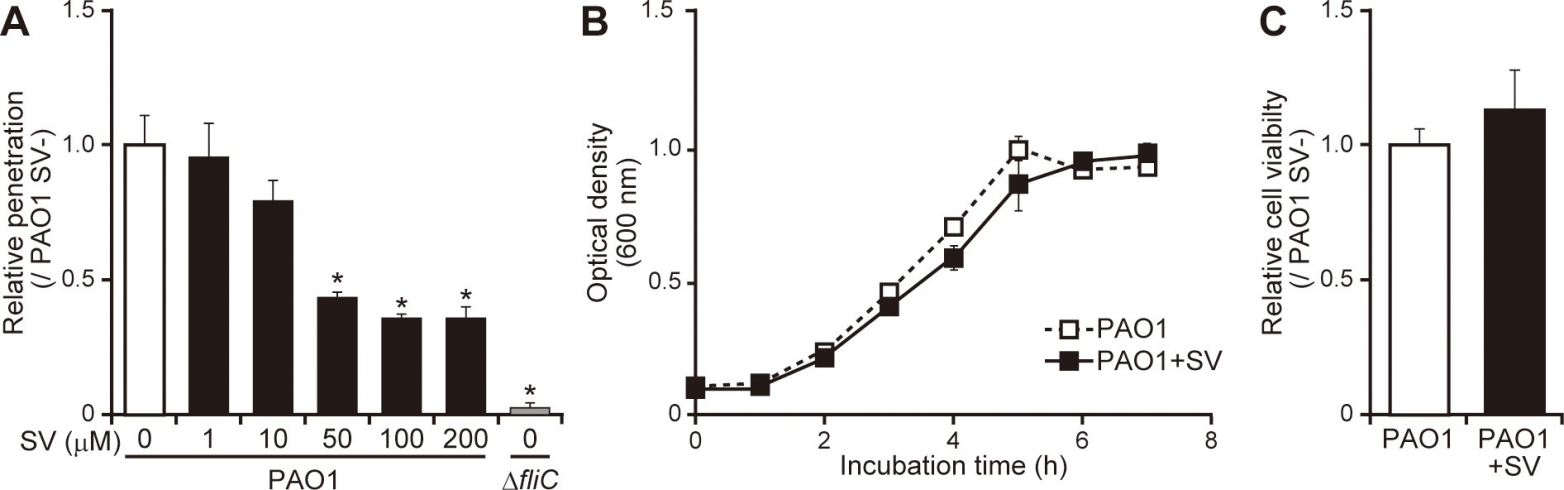
Sulfated vizantin suppresses *P*. *aeruginosa* mucin penetration without influencing *P*. *aeruginosa* growth. (A) Transwell chambers were filled with the indicated concentrations of sulfated vizantin (SV). After addition of *P*. *aeruginosa* PAO1 or Δ*fliC* to the top chamber, the number of bacteria in the bottom chamber was counted. The graph shows penetration relative to that of PAO1 in the absence of SV, with the data representative of five separate experiments. Error bars indicate standard error (*n* = 5). *P < 0.05 as compared with PAO1 in the absence of SV. (B) *P*. *aeruginosa* PAO1 was incubated with 100 μM SV or without SV, and after incubation for the indicated times, the OD_600_ of the culture was measured. The data are representative of three separate experiments. Error bars indicate standard error (*n* = 3). (C) *P*. *aeruginosa* PAO1 was incubated with 100 μM SV, and after incubation for 3 h, CFUs were counted to quantify the viable bacteria. The graph shows viable cells relative to that of PAO1 in the absence of SV, with the data representative of five separate experiments. Error bars indicate standard error (*n* = 5).

To determine whether sulfated vizantin inhibits mucin penetration of *P*. *aeruginosa*, we performed an artificial mucin penetration assay. After a 3 h incubation, we observed a 2.3-, 2.8-, and 2.8-fold decrease in the number of *P*. *aeruginosa* colonies in the presence of sulfated vizantin (50, 100, and 200 μM, respectively) in the bottom chamber (Fig 1A; *P* < 0.05), whereas there was no difference in the number of *P*. *aeruginosa* colonies in the bottom chamber exposed to sulfated vizantin (1 and 10 μM) as compared with untreated cells (Fig 1A; *P* > 0.05). As previously reported [8,22], deletion of the *fliC* gene significantly decreased the number of *P*. *aeruginosa* colonies in the bottom chamber (Fig 1A; *P* < 0.05). Additionally, non-sulfated vizantin (100 μM) decreased the penetration of *P*. *aeruginosa* PAO1 through the mucin layer (S2 Appendix; *P* < 0.05). Furthermore, sulfated vizantin (100 μM) decreased the penetration of *P*. *aeruginosa* clinical strains through the mucin layer (S3 Appendix; *P* < 0.05). To determine whether sulfated vizantin affected the growth of *P*. *aeruginosa*, we measured the viability *P*. *aeruginosa* cells after treatment with sulfated vizantin. Results showed that sulfated vizantin (100 μM) did not significantly decrease bacterial growth within the detection time until 7 h after inoculation (Fig 1B; *P* > 0.05). Additionally, sulfated vizantin (100 μM) did not significantly decrease bacterial cell viability at 3 h after inoculation (Fig 1C; *P* > 0.05).

### Sulfated vizantin inhibits the flagella-dependent motility of *P*. *aeruginosa*

**Fig 2:**
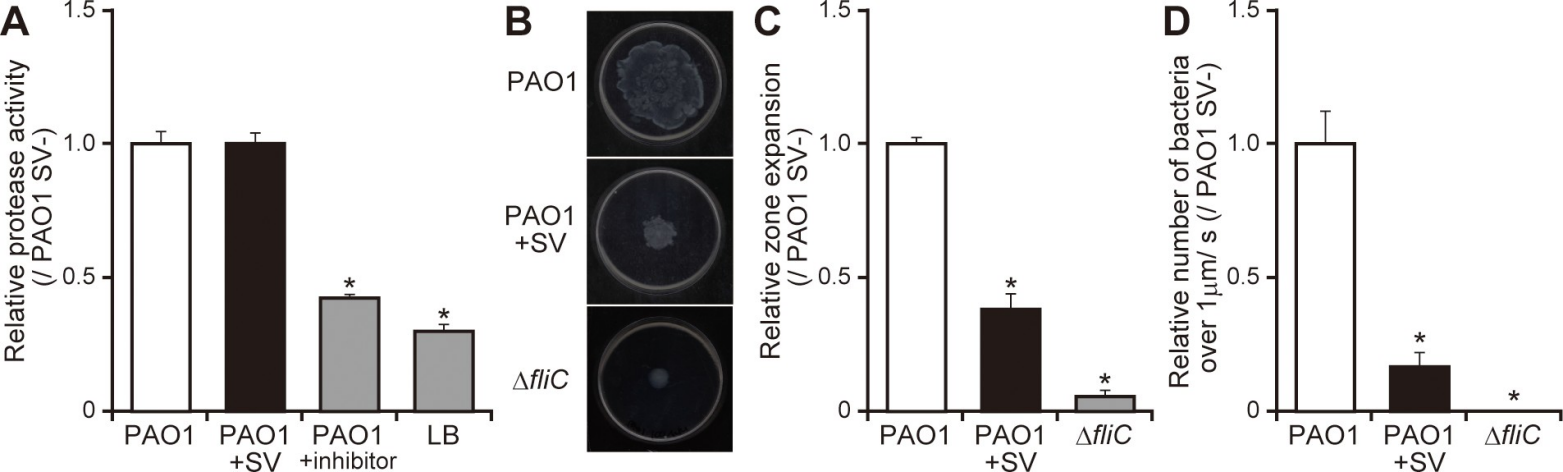
Sulfated vizantin inhibits the flagellar-dependent motility of *P*. *aeruginosa*. (A) After incubation of *P*. *aeruginosa* PAO1 with 100 μM sulfated vizantin (SV) or without SV, as well as PAO1 with protease inhibitors or LB broth only (control), azocasein-degradation activity was measured. The data are representative of three separate experiments, and the graph shows degradation activity relative to that of PAO1 in the absence of SV. Error bars indicate standard error (*n* = 3). *P < 0.05 as compared with PAO1 in the absence of SV. (B) *P*. *aeruginosa* PAO1 or Δ*fliC* was spotted on swimming agar plates with 100 μM SV or without SV, and after incubation for 12 h, the plates were observed and photographed. Typical plates are shown. (C) Measurement of the diameter distance of the swimming zone. The graph shows the distance of the swimming zone relative to that of PAO1 in the absence of SV, and the data are representative of three separate experiments. Error bars indicate standard error (*n* = 3). *P < 0.05 as compared with PAO1 in the absence of SV. (D) After incubation of *P*. *aeruginosa* PAO1 or Δ*fliC* with 100 μM SV or without SV for 3 h, bacterial cultures were mixed with 0.5% GTG agarose gel and loaded onto a glass slide. Bacterial cells were recorded as a movie using an EVOS microscope, and moving speed was calculated from the distance that the bacterial moved over 20 s, with the number of bacteria moving >1 μm/s determined using the MTrackJ plugin in ImageJ software. Cell movement >1 μm/s was measured in five fields, with the number of bacteria per field ~30 cells. The graph shows the number of bacteria moving >1 μm/s relative to PAO1 in the absence of SV, and the data are representative of five separate experiments. Error bars indicate standard error (*n* = 5). *P < 0.05 as compared with PAO1 in the absence of SV.

We previously demonstrated that *P*. *aeruginosa* PAO1 penetrates the mucin layer by flagellar motility and a mucin degrading protease [8,22]. In the present study, sulfated vizantin (100 μM, 3 h incubation) did not significantly decrease bacterial protease activity (Fig 2A; *P* > 0.05), although protease inhibitors significantly decreased bacterial protease activity in *P*. *aeruginosa* (Fig 2A; *P* < 0.05). Additionally, we observed a 2.6-fold decrease in the swimming zone of *P*. *aeruginosa* in the presence of sulfated vizantin (100 μM, 3 h incubation) (Fig 2B and 2C; *P* < 0.05). As previously reported [3], deletion of the *fliC* gene significantly decreased the swimming zone of *P*. *aeruginosa* on soft agar plates (Fig 2B and 2C; *P* < 0.05). Moreover, observations using a cell-tracking system showed that there was a 6.0-fold decrease in the number of *P*. *aeruginosa* cells that moved >1 μm/s in the presence of sulfated vizantin (100 μM, 3 h incubation) (Fig 2D and S5 and S6 Appendices; *P* < 0.05). Moreover, deletion of the *fliC* gene diminished the number of moving cells (Fig 2D and S7 Appendix; *P* < 0.05). In our observations (Fig 2D and S5 and S6 Appendices), there are two reasons considered to explain decreases in the number of motile *P*. *aeruginosa* cells: 1) *P*. *aeruginosa* cells were trapped by the 0.5% GTG agarose gel, and 2) *P*. *aeruginosa* cells were attached on the glass slide. Furthermore, we found no significant differences in the number of bacteria on glass slides among all conditions.

### Sulfated vizantin does not alter the flagellar composition of *P*. *aeruginosa*

**Fig 3:**
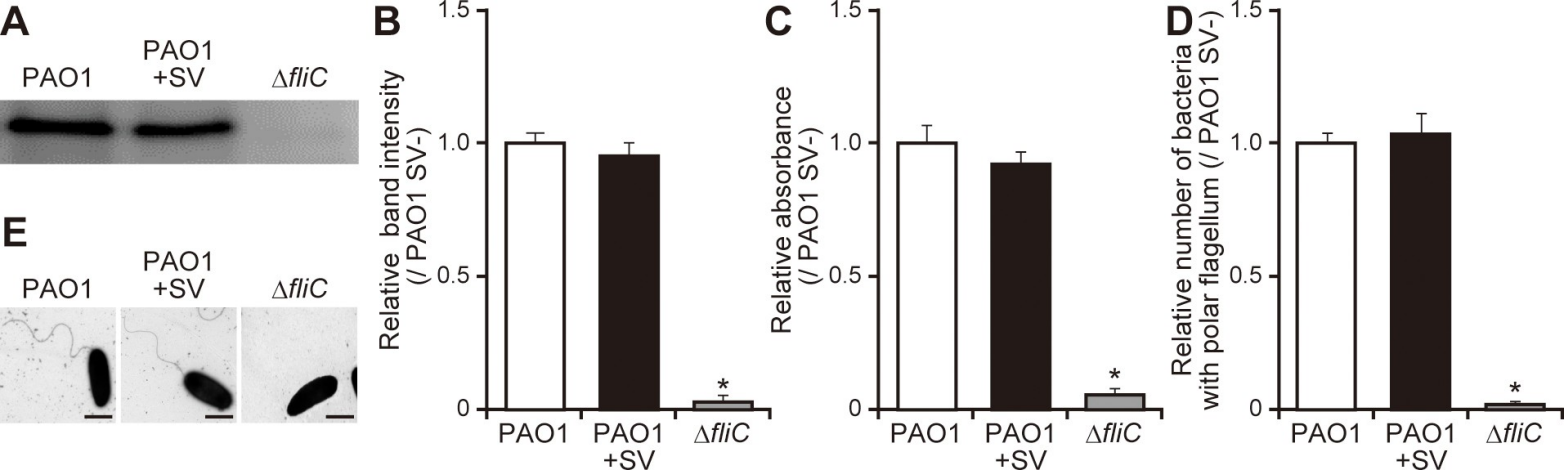
Influence of a flagellar filament in *P*. *aeruginosa* treated with sulfated vizantin. (A) After incubation of *P*. *aeruginosa* PAO1 or Δ*fliC* with 100 μM sulfated vizantin (SV) or without SV for 3 h, total FliC protein in *P*. *aeruginosa* was determined by western blot using an anti-FliC antibody. Typical images are shown. (B) Band intensities were measured using ImageJ software. The graph shows band intensity relative to that of PAO1 in the absence of SV, and the data are representative of three separate experiments. Error bars indicate standard error (*n* = 3). *P < 0.05 as compared with PAO1 in the absence of SV. (C) After incubation of *P*. *aeruginosa* PAO1 or Δ*fliC* with 100 μM SV or without SV for 3 h, total surface FliC protein in *P*. *aeruginosa* was determined by ELISA using an anti-FliC antibody. The graph shows absorbance relative to that of PAO1 in the absence of SV, and the data are representative of three separate experiments. Error bars indicate standard error (*n* = 3). *P < 0.05 as compared with PAO1 in the absence of SV. (D) After incubation of *P*. *aeruginosa* PAO1 or Δ*fliC* with 100 μM SV or without SV for 3 h, bacterial cultures were loaded onto a glass slide, and bacterial cells underwent Leifson staining. Visualization of the flagellar filament in treated and untreated *P*. *aeruginosa* was performed using light microscopy, with cells measured in five fields. Number of bacteria per field was ~50 cells. The graph shows the number of bacteria with a single polar flagellum relative to that of PAO1 in the absence of SV, and the data are representative of five separate experiments, and the error bars indicate standard error (*n* = 5). *P < 0.05 as compared with PAO1 in the absence of SV. (E) After incubation of *P*. *aeruginosa* PAO1 or Δ*fliC* with 100 μM SV or without SV for 3 h, visualization of the flagellar filament in treated and untreated *P*. *aeruginosa* was performed using TEM. Scale bars, 1 μm. Typical images are shown.

Our findings demonstrated that sulfated vizantin decreased flagellar motility (Figs 1A and 2B–2D). To clarify the mechanism by which sulfated vizantin decreases the flagellar motility of *P*. *aeruginosa*, we examined FliC protein levels in *P*. *aeruginosa* cells in the presence or absence of sulfated vizantin (100 μM, 3 h incubation). We found no differences in the amounts of FliC protein between bacteria in the presence or absence of sulfated vizantin by western blot analysis (Fig 3A and 3B; *P* > 0.05). Additionally, there was no difference in FliC protein levels on the surface of *P*. *aeruginosa* cells exposed to sulfated vizantin (100 μM, 3 h incubation) as compared with untreated cells according to ELISA (Fig 3C; *P* > 0.05). Next, to observe the number of *P*. *aeruginosa* cells with a single polar flagellum, we performed Leifson staining of *P*. *aeruginosa* cells in the presence or absence of sulfated vizantin (100 μM, 3 h incubation). We observed that 74% [standard division ± 12.5% (*n* = 50), five fields] of *P*. *aeruginosa* cells without sulfated vizantin showed single polar flagellum, whereas 77% [standard division ± 10.9% (*n* = 50), five fields] of *P*. *aeruginosa* cells with sulfated vizantin showed a single polar flagellum. There was no significant differences in the number of bacteria with a single polar flagellum as compared with untreated cells according to Leifson staining (Fig 3D; *P* > 0.05). Furthermore, there were no differences in flagellar-filament morphology observed on the surface of *P*. *aeruginosa* cells, regardless of the presence or absence of sulfated vizantin (100 μM, 3 h incubation), according to TEM (Fig 3E).

### Sulfated vizantin inhibits flagella-related gene expression in *P*. *aeruginosa*

**Fig 4:**
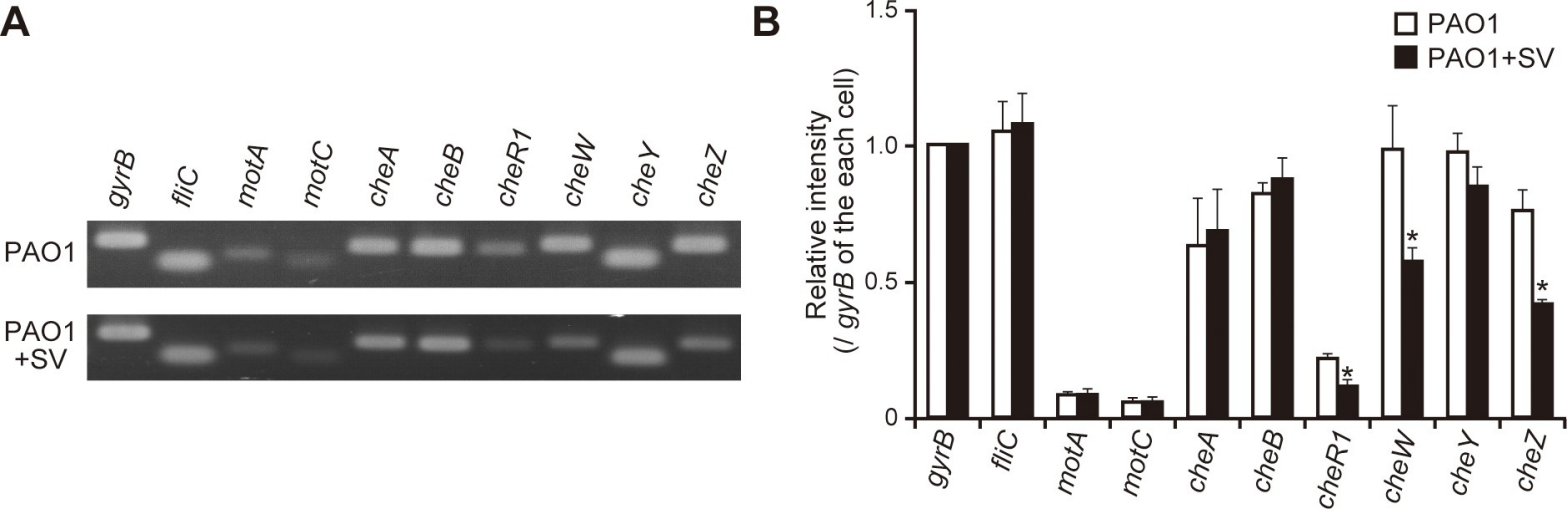
Effects of sulfated vizantin on the mRNA expression of flagellar genes in *P*. *aeruginosa*. (A) After incubation of *P*. *aeruginosa* PAO1 with 100 μM sulfated vizantin (SV) or without SV for 3 h, mRNA expression of the indicated genes was determined by RT-PCR. Typical gel images are shown. (B) Band intensities were measured using ImageJ software, and the *gyrB* gene was used as an internal control. The graph shows band intensity relative to *gyrB* expression in each cell, and the data are representative of five separate experiments. Error bars indicate standard error (*n* = 5). *P < 0.05 as compared with PAO1 in the absence of SV.

We then examined the effects of sulfated vizantin (100 μM, 3 h incubation) on mRNA expression of the flagella-filament gene (*fliC*), motor genes (*motA* and *motC*), and chemotaxis genes (*cheA*, *cheB*, *cheR*1, *cheW*, *cheY*, and *cheZ*). After a 3 h incubation, we observed a 1.9-, 1.8-, and 1.7-fold decrease in the mRNA expression of *cheR1*, *cheW*, and *cheZ*, respectively, in *P*. *aeruginosa* in the presence of sulfated vizantin (Fig 4A and 4B; *P* < 0.05). By contrast, sulfated vizantin did not significantly decrease the mRNA expression of *fliC*, *motA*, *motC*, *cheA*, *cheB*, and *cheY* in *P*. *aeruginosa* PAO1 (Fig 4A and 4B; *P* > 0.05).

## Discussion

In this study, we examined whether sulfated vizantin inhibited the flagellar motility of *P*. *aeruginosa* PAO1. Our results demonstrated that sulfated vizantin suppressed penetration of *P*. *aeruginosa* PAO1 through an artificial mucin layer and inhibited swimming motility without influencing the growth rate of *P*. *aeruginosa* PAO1 or influencing protease activity. Additionally, sulfated vizantin did not affect the composition of the flagellar filament in *P*. *aeruginosa* PAO1; however, it decreased mRNA expression of the chemotaxis-related genes *cheR*1, *cheW*, and *cheZ*. Collectively, our results showed that sulfated vizantin inhibited flagella of *P*. *aeruginosa* PAO1 by suppressing the chemotactic system.

The recent emergence of MDR *P*. *aeruginosa* represents a potentially major public health problem [23–25]. Instead of current treatments, such as antibiotics that kill or inhibit the growth of this bacterium, new types of agents that antagonize specific virulence factors are required as novel therapeutic targets. Therefore, the many *P*. *aeruginosa* virulence determinants, including type III secretion [33,34], quorum sensing [35], biofilm formation [36], and flagella [18], have been the focus of much investigation [37]. Findings from several animal models demonstrated that antibodies against the flagellum induce either active or passive prevention of *P*. *aeruginosa* infection in the lung [17,38] and urinary tract [20]. Moreover, a randomized placebo-controlled trial of patients with cystic fibrosis found a statistically significant reduction in infection episodes over a 2-year period in those immunized with a bivalent flagella vaccine [39]. These studies suggest that the flagella-dependent motility of *P*. *aeruginosa* represents a potential target for anti-infective drugs. In this study, our data showed that sulfated vizantin suppressed swimming motility without impairing the growth capacity of *P*. *aeruginosa*. Collectively, our findings indicated that sulfated vizantin might be of potential use in the prevention and treatment of flagella-related infections. However, further studies are needed to investigate whether sulfated vizantin possesses any synergistic effects on *P*. *aeruginosa* infection using animal infection models, such as those involving sepsis.

Our results showed that sulfated vizantin suppressed swimming motility dependent on flagella without influencing the growth rate or protease activity of *P*. *aeruginosa* PAO1. Researchers have suggested three main elements that regulate flagellar motility: 1) flagellar filament structure [40], 2) motor complex [12], and 3) chemotactic signal transduction [13,16]. Because we found no significant differences in FliC protein levels and observed no changes in flagellar filament structure between bacteria treated or untreated with sulfated vizantin, we propose that sulfated vizantin does not influence the composition of the flagellar filament structure. This was also supported by observations that sulfated vizantin did not affect the mRNA expression of genes encoding proteins involved in the motor complex. Chemotactic signal transduction includes transmembrane receptors (MCPs), the receptor-coupled histidine kinase CheA, the linker CheW, the response regulator CheY, and the phosphatase CheZ (S4 Appendix) [13,16]. Upon binding to certain attractants, an MCP transmits the signal to CheA [13,16], which in turn acts as a phosphodonor for CheY [13,16], followed by phosphorylated CheY interacting with the rotational-switch protein in flagellar motors [13,16]. Additionally, the opposing activities of two specific enzymes (CheR and CheB; a methyltransferase and methylesterase, respectively) control MCP methylation levels [13,16]. On the other hand, CheY phosphorylation relies upon the activity of CheA, and its dephosphorylation is controlled by CheZ, as well as by intrinsic dephosphorylation activity [13,16]. Furthermore, the mRNA expression of many genes involved in flagellar filament structure, motor complex, chemotactic signal transduction, and energy metabolism controls bacterial motility by affecting flagella activity [13,16]. In the present study, our findings showed that sulfated vizantin decreased the mRNA expression of *cheR*1, *cheW*, and *cheZ* by ~50%. Importantly, deletion of these genes diminishes swimming motility of *P*. *aeruginosa* [13,16,41]. Based on these findings, we propose that sulfated vizantin decreases *P*. *aeruginosa* flagellar motility by disturbing chemotactic signal transduction. Further studies are needed to elucidate the detailed mechanisms associated with suppression of chemotactic signal transduction by sulfated vizantin.

Bacterial flagellar motility represents a key virulence factor in enteric bacteria, such as *E*. *coli*, *Salmonella enterica* serovar Typhimurium, and *Proteus mirabilis* [42–44]. The present study focused on *P*. *aeruginosa* PAO1; however, the role of sulfated vizantin should be investigated in MDR *P*. *aeruginosa* and other species in future studies. Collectively, we demonstrated that sulfated vizantin is an effective inhibitor of flagellar motility in *P*. *aeruginosa* PAO1 *in vitro* and might be of potential use in the prevention and treatment of flagella-related infections, such as pneumonia, urinary tract infection, and bacteremia [17–20].

## Supporting information

S1 Appendix Structure of sulfated vizantin. (TIF)

S2 Appendix Non-sulfated vizantin suppresses *P*. *aeruginosa* mucin penetration. Transwell chambers were filled with 100 μM sulfated vizantin (SV), 100 μM non-sulfated vizantin, or saline (control). After addition of *P*. *aeruginosa* PAO1 to the top chamber, the number of bacteria in the bottom chamber was counted. The graph shows penetration relative to that of PAO1 in the absence of SV, and the data are representative of five separate experiments. Error bars indicate standard error (*n* = 5). *P < 0.05 as compared with PAO1 in the absence of SV.
(TIF)

S3 Appendix Sulfated vizantin suppresses mucin penetration by some *P*. *aeruginosa* strains. Transwell chambers were filled with 100 μM sulfated vizantin (SV) or saline (control). After addition of *P*. *aeruginosa* PAO1, ATCC10145, or ATCC25619 to the top chamber, the number of bacteria in the bottom chamber was counted. The graph shows penetration relative to that of each strain in the absence of SV, and the data are representative of five separate experiments. Error bars indicate standard error (*n* = 5). *P < 0.05 as compared with each strain in the absence of SV.
(TIF)

S4 Appendix The chemotaxis pathways in *P*. *aeruginosa* PAO1. (TIF)

S5 Appendix The motility of *P*. *aeruginosa* PAO1 in the absence of sulfated vizantin. After incubation of *P*. *aeruginosa* PAO1 for 3 h, bacterial cultures were mixed with 0.5% GTG agarose gel and loaded onto a glass slide. Movement of bacterial cells was recorded using an EVOS microscope over 20 s. A typical movie of this activity is shown.
(AVI)

S6 Appendix The motility of *P*. *aeruginosa* PAO1 in the presence of sulfated vizantin. After incubation of *P*. *aeruginosa* PAO1 with 100 μM sulfated vizantin (SV) or without SV for 3 h, bacterial cultures were mixed with 0.5% GTG agarose gel and loaded onto a glass slide. Movement of bacterial cells was recorded using an EVOS microscope over 20 s. A typical movie of this activity is shown.
(AVI)

S7 Appendix The motility of *P*. *aeruginosa* Δ*fliC* in the absence of sulfated vizantin. After incubation of *P*. *aeruginosa* Δ*fliC* for 3 h, bacterial cultures were mixed with 0.5% GTG agarose gel and loaded onto a glass slide. Movement of bacterial cells was recorded using an EVOS microscope over 20 s. A typical movie of this activity is shown.
(AVI)
