# Supplementary figures and images for: Sulfated vizantin suppresses mucin layer penetration dependent on the flagella motility of Pseudomonas aeruginosa PAO1
Source: PLoS One. 2018 Nov 1;13(11):e0206696. doi: 10.1371/journal.pone.0206696 (PMC6211736; doi:10.1371/journal.pone.0206696)

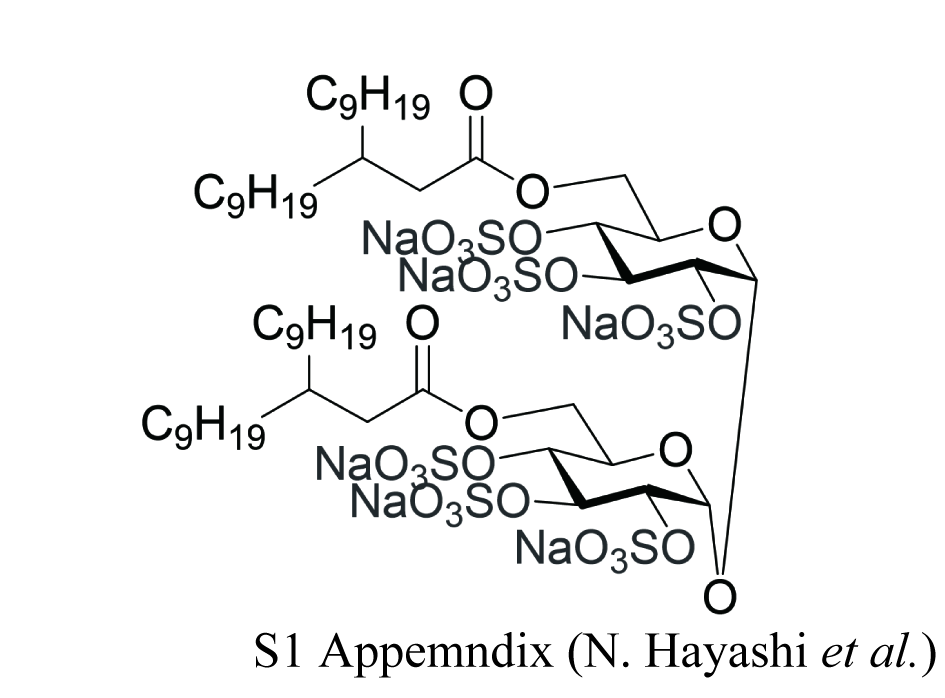

Supplement: S1 Appendix — (TIF) [file pone.0206696.s001.tif]

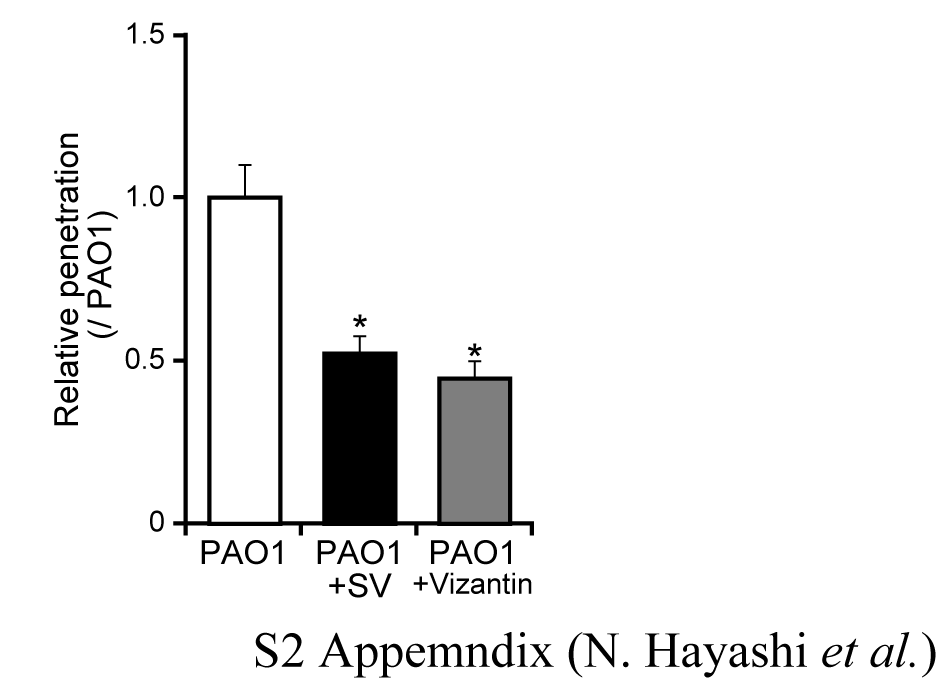

Supplement: S2 Appendix — Transwell chambers were filled with 100 μM sulfated vizantin (SV), 100 μM non-sulfated vizantin, or saline (control). After addition of P. aeruginosa PAO1 to the top chamber, the number of bacteria in the bottom chamber was counted. The graph shows penetration relative to that of PAO1 in the absence of SV, and the data are representative of five separate experiments. Error bars indicate standard error (n = 5). *P < 0.05 as compared with PAO1 in the absence of SV. (TIF) [file pone.0206696.s002.tif]

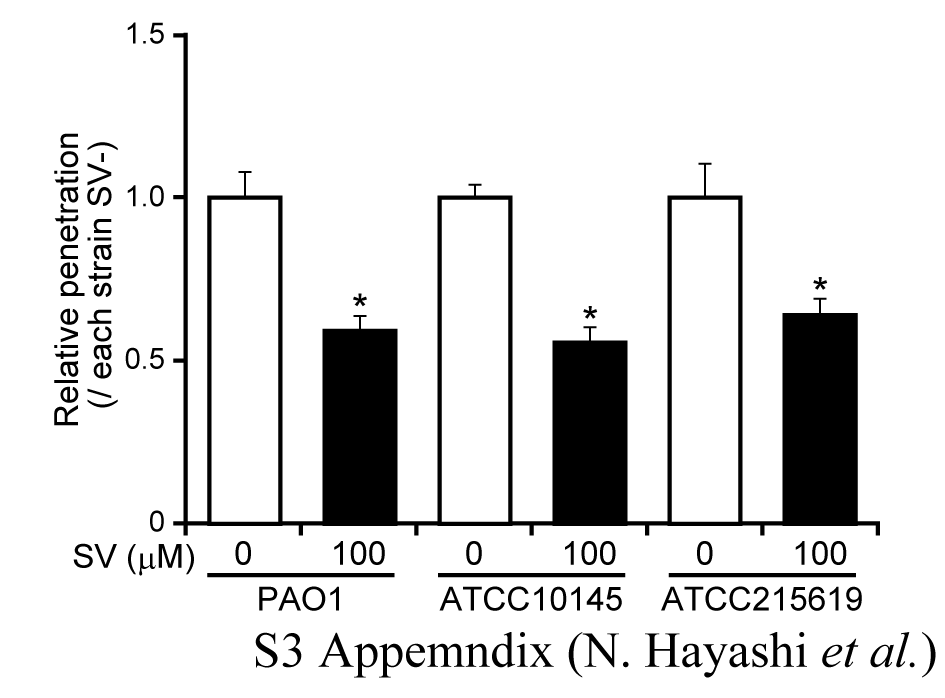

Supplement: S3 Appendix — Transwell chambers were filled with 100 μM sulfated vizantin (SV) or saline (control). After addition of P. aeruginosa PAO1, ATCC10145, or ATCC25619 to the top chamber, the number of bacteria in the bottom chamber was counted. The graph shows penetration relative to that of each strain in the absence of SV, and the data are representative of five separate experiments. Error bars indicate standard error (n = 5). *P < 0.05 as compared with each strain in the absence of SV. (TIF) [file pone.0206696.s003.tif]

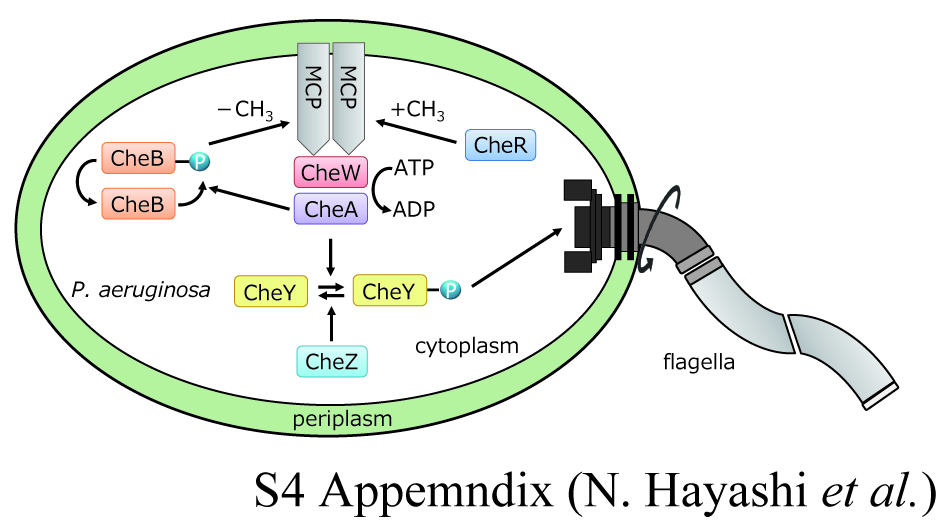

Supplement: S4 Appendix — (TIF) [file pone.0206696.s004.tif]
